# The MIR 2018 Exam: Psychometric Study and Comparison with the Previous Nine Years

**DOI:** 10.3390/medicina55120751

**Published:** 2019-11-20

**Authors:** Jaime Baladrón, Fernando Sánchez Lasheras, José María Romeo Ladrero, Tomás Villacampa, José Curbelo, Paula Jiménez Fonseca, Alberto García Guerrero

**Affiliations:** 1 Curso Intensivo MIR Asturias, c/ Quintana 11A, 33005 Oviedo, Spain; jaime@baladron.com; 2 Mathematics Department, Faculty of Sciences, University of Oviedo, c/ Federico García Lorca 18, 33007 Oviedo, Spain; 3 Blog GangasMIR, c/ Coso 126, 50002 Zaragoza, Spain; gangasmir@gmail.com; 4 Villacampa Ophthalmic Clinic, c/ La Cámara 15, 33401 Avilés, Spain; tomas@villacampa.net; 5 Internal Medicine Department, Hospital Universitario La Princesa, 28006 Madrid, Spain; curbelo1984@gmail.com; 6 Medical Oncology Department, Hospital Universitario Central de Asturias, 33011 Oviedo, Spain; palucaji@hotmail.com; 7 Cardiology Department, Hospital San Agustín, 33401 Avilés, Spain; albertogarciaguerrero@hotmail.com

**Keywords:** classical test theory (CTT), educational evaluation, item response theory (IRT), medical students, psychometry, specialization, statistics

## Abstract

*Background and Objectives*: The aim of the present research is to study the questions used in the 2018 MIR exam (a test that allows access to specialized medical training in Spain), describe their psychometric properties, and evaluate their quality. *Materials and Methods*: This analysis is performed with the help of classical test theory (CTT) and item response theory (IRT). The answers given to the test questions by a total of 3868 physicians are analyzed. *Results*: According to CTT, the average difficulty index for all of the test questions was 0.629, which falls into the acceptable category. The average difficulty index with correction for random effects was 0.515, which corresponds to a value within the optimal range. The mean discrimination index was 0.277, which is in the good category, while the mean point biserial correlation coefficient, with a value of 0.275 fits in the regular category. The values of difficulty and discrimination calculated according to the model of two parameters of the IRT seem adequate with average values of −0.389 and 0.677. The Cronbach alpha score obtained for the overall test was 0.944. This value is considered as very good. *Conclusions*: A decrease was observed in the average values of discrimination in the last three calls, which may be related to the greater proportion of Spanish graduates that take the exam in the same year of finalization of their studies in Medicine.

## 1. Introduction

In Spain, in order to practice as a medical professional, in addition to having a degree in Medicine, it is necessary to have a specialist degree. All the degrees and Master’s programs offered by universities in Spain must be approved by ANECA, (Agencia Nacional de Evaluación de la Calidad y Acreditación), an autonomous organization affiliated to the Ministry of Science, Innovation and Universities. All of the Medicine faculties in Spain use study plans that are based on directive RD1417/1990. This directive details the subjects to be studied and the number of hours required for each of these. A maximum and a minimum number of hours is specified, which gives a certain degree of freedom to each center. At the end of their studies, after six academic years, students receive a degree that is equivalent to a Bachelor’s plus a Master’s degree. With this degree, they are able to apply for a specialized medical training position. Spain belongs to the European Higher Education Area, which currently comprises a total of 48 countries, and degrees in Medicine, like other university degrees, are in line with those in other countries in the Area.

Since 1978 [1], access to specialized medical training in Spain has been done through the MIR test [2,3,4,5,6]. This test is convened by the Ministries of Health and Education and is held annually. In the case of the 2018 call, this was made by means of Order SCB/947/2018 of 7 September and was published in the Official State Gazette on 14 September 2018.

From 2009 to 2018, the exam has comprised a total of 235 questions, of which the last 10 are reserve questions. They belong to different subjects and cover all specialties of Medicine. One of the weaknesses of the test is that these questions have still not been calibrated. There is a technical committee that reviews the claims of exam candidates about different questions after the test, and it can cancel questions that are either potentially incorrect or confusing. One of the strengths of the MIR exam is that it is transparent, thus all the stakeholders know the rules and how it is scored.

The aim of the MIR test is to allow the ordination of the candidates in a list that permit the selection of medical specialty. The selection is performed considering both the marks obtained by the candidate in their degree (weighted value of 10%) and the result of the exam (weighted value of 90%). Specialty selection is performed according to the total marks obtained, starting with the highest and then followed in decreasing order. Due to the great importance of this test for the future of the exam candidates, they put a great deal of effort and attention into studying for it when they start their sixth and last year of the degree.

The number of candidates admitted to the test in 2018 was 15,519, of whom 14,187 took the exam and opted for one of the 6797 places available. Of those taking the exam, 10,273 were Spanish doctors (72.41%) and 3914 were foreign physicians (27.59%). In 2018, the cut-off grade was 65 net points. The net points are calculated by subtracting one-third of the wrong questions from the total number of correct answers. The cut-off point led to the direct elimination of 2983 applicants (21.03%), leaving them with no chance at all to choose a place for their specialized training.

Once the test has been passed, the specialty training programs can be accessed in different hospitals all around the country and are relatively homogeneous. All hospitals in which the training is conducted must undergo periodic evaluations to maintain their accreditation. In the rest of the countries of Europe, there is great variability in the form of access to specialized medical training, thus, it is currently not possible to speak of a common European model of access to medical specialties.

In this study, the test questions were analyzed with the help of both classical test theory (CTT) [7] and item response theory (IRT) [8] and then compared to the metrics of previous years’ exams. It should be noted that while CTT is based on the evaluation of the accuracy and reliability of the test measurement, IRT focuses on studying the performance of a set of individuals before a test and determining how it is possible to measure the relationship between a hidden trait (knowledge of the subject matter of the evaluation) and the probability of subjects answering the proposed questions correctly. This relationship is given by the item response function [8].

## 2. Materials and Methods

### 2.1. The Examination Under Study

As in the previous test from 2009 onwards, the MIR 2018 exam was comprised of a total of 235 questions, of which the last 10 were reserve questions. Since five were cancelled, the number of questions under consideration in this study was 230.

### 2.2. Database

The answers given to the different test question were provided by a total of 3868 participants, who voluntarily entered them into an ad hoc web application developed by the company Cursos Intensivos MIR Asturias (Oviedo, Spain). This sample represents 27.31% of the total number of applicants who took the test all around the country. The task of entering the answers for the complete test into the web application took from 10 to 20 min. It is possible that mistakes could be made in the process of entering the answers. However, we believe that there were probably few errors. As they were able to obtain their mark in advance by participating in the study, it is likely that they provided accurate information. It was possible to amend an answer selected in case of error. Please note that participants were not recruited in a formal way, anybody who had performed the test and wanted to know his/her mark in advance of the official results, was able to introduce their answers on the web.

### 2.3. Data Analysis

#### 2.3.1. Reliability

Since the purpose of the MIR exam is to rank doctors depending on their knowledge, and to enable access to their choice of a specialized training place, it is of interest to find out how reliable this test really is. The reliability of a test is defined as the consistency with which it is able to measure a given variable. The determination of this reliability was made by means of the coefficient α proposed by Cronbach. It is expressed as follows [7],

(1) $$\alpha =\frac{K}{K-1}\cdot (1-\frac{\sum_{j=1}^{n}\sigma_{j}^{2}}{\sigma_{x}^{2}})$$

where K is the total number of questions in the test, $\overset{n}{\underset{j=1}{\sum }}\sigma_{j}^{2}$ is the sum of the variances of the *n* items for each question, and $\sigma_{x}^{2}$ is the variance of the total test scores.

Cronbach’s α values are between 0 and 1. The closer to 1, the more reliable the test will be.

#### 2.3.2. Difficulty Index

The difficulty index of an item is defined as the proportion of individuals who match it out of all those who take the test. It is expressed by means of the following formula [7,9]:

(2) $$D=\frac{A}{N}$$

where A is the number of individuals who answer the question correctly., and N is the number of individuals who submit the test.

#### 2.3.3. Difficulty Index with Correction of Random Effects

The calculation of the difficulty index with correction for the effects of chance was carried out with the help of the following formula [10]:

(3) $$ID=\frac{A-\frac{E}{K-1}}{N}$$

where A is the number of subjects who answer the item correctly; E is the number of subjects who incorrectly answer the item; K is the number of response alternatives to the item, four in the case of this study; and N is the total number of participants in the test.

#### 2.3.4. Discrimination Index

The discriminatory capacity of a question is of great importance in determining its contribution to the management of the individuals being tested. For the purposes of this paper, the following index was used [10]:

(4) $$DS=2\cdot \frac{F-D}{N_{1}+N_{2}}$$

where F is the number of correct answers in the strong group, D is the number of correct answers in the weak group, $N_{1}$ is the number of individuals answering the question in the strong group, regardless of whether they are right or wrong, and $N_{2}$ is the number of individuals who answered the question in the weak group, regardless of whether or not they got it right.

The strong group is defined as the 27% of individuals who answered the greatest number of questions in the test correctly, and the weak group is defined as the 27% of individuals who answered the least number of questions correctly.

#### 2.3.5. Point Biserial Correlation Index

The point biserial correlation index relates the global result of the test for the subjects who get the analyzed question right to that of those who get it wrong [8]. The formula used was [7]

(5) $$\rho_{bp}=\frac{\mu_{p}-\mu_{q}}{\sigma_{x}}\sqrt{\frac{ID}{1-ID}}$$

where $\mu_{p}$ is the average test score of the subjects who answer the item correctly, $\mu_{q}$ is the average test score of subjects who fail to answer the item correctly, $\sigma_{x}$ is the standard deviation of the total test score, and ID is the difficulty index of the item defined as the number of subjects who answer the item correctly versus the total number of subjects taking the test.

#### 2.3.6. The Two Parameter Model of IRT

The main usefulness of IRT lies in its ability to predict the probability of success of the exam participants in the questions to which they are exposed, according to their level of knowledge. From the experience acquired by the authors in previous studies [11,12,13], it was decided to make use of the logistic model of two parameters (2PL) [8]:

(6) $$\left(\mu j=1|\theta_{i},a_{j},b_{j}\right)=\frac{e^{1,7a_{j}\left(\theta_{i}-b_{j}\right)}}{1+e^{1,7a_{j}\left(\theta_{i}-b_{j}\right)}}$$

where $\theta_{i}$ is the level of knowledge of the ith subject examined, $a_{j}$ is the discrimination value of the j-th question, and $b_{j}$ is the difficulty level of the j-th question.

The probability of a certain individual getting a question right depends as much on the characteristics of the question as on the level of knowledge of the individual.

Table 1 presents an assessment of the difficulty indices by category. The categorization for the difficulty index, the difficulty index with correction of random effects, the discrimination index, and the point biserial correlation index correspond to a well-known classification [7] that has been used by authors in previous studies [7,9,14]. Finally, the categories of the IRT discrimination index were proposed by the authors in a previous paper [14].

## 3. Results

### 3.1. Analysis of Exam Questions

The overall reliability of the examination was assessed using the Cronbach alpha coefficient, whose value was 0.944. This value can be considered as very good. 

**Figure 1:**
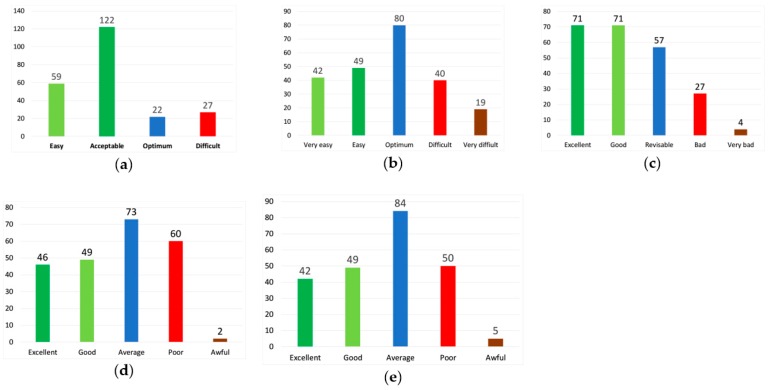
Distribution of questions by category: (**a**) difficulty index, (**b**) difficulty index with correction of the effects of chance, (**c**) discrimination index, (**d**) point biserial correlation index, (**e**) item response theory (IRT) discrimination index.

Figure 1 shows the distribution by category of the test questions according to difficulty and discrimination. With regard to the difficulty index, the majority of the questions (53.04%) fell within the category considered acceptable. For the difficulty index, which took into account the correction of the effects of chance, 80 questions, 34.78%, belonged to the category considered as optimal. The extreme categories (very easy and very difficult) accounted for only 26.52% of the total number of questions analyzed. One hundred and forty-four questions, 61.74%, had a discrimination index that is considered good or excellent. Regarding the point biserial correlation coefficient, 95 questions (41.30%) were considered to be good or excellent, 73 questions (31.74%) belonged to the regular category, the most frequent category for this index, and there are 62 questions that were classified as poor or bad (26.96%). The classification of the questions according to the IRT discrimination index was quite similar to that obtained by means of the point biserial correlation, where 39.57% of the questions were classified as good or excellent, 36.52% of the questions were classified as regular, and 23.91 % were classified as poor or awful.

### 3.2. Analysis of Exam Questions Grouped by Subject

The MIR 2018 exam questions refer to a total of 36 subjects grouped in three blocks (see Table 2 and Table 3). The Systems (associated with medical and surgical specialties) block contained the greatest number of questions in this test with 46.52%, followed by Other specialties with 42.18%, and finally, Basic subjects with 11.30%.

Table 2 and Table 3 show the average indices of difficulty, difficulty with correction of random effects, discrimination, and point biserial correlation as well as the indices of difficulty and discrimination calculated according to the two-parameter model of IRT, for each Subject.

When we analyzed the average difficulty index of the questions divided by subject, we observed that there were four that could be classified as easy (Communicative Skills, Bioethics, Anesthesiology, and Genetics), nine that, on average, presented optimal difficulty (Emergencies, Palliative, Preventive, Pediatrics, Oncology, Pharmacology, Immunology, Clinical Management, and Infectious Diseases) as well as only one subject that was considered difficult (Vascular Surgery). The remaining 22 subjects presented an acceptable average level of difficulty.

Regarding the average difficulty index with correction of the random effects, two of the subjects were classified as very easy (Communicative Skills and Bioethics), three as easy (Anesthesiology, Genetics and Ophthalmology), five as difficult (Anatomy, Dermatology, Pathological Anatomy, Geriatrics, and Vascular Surgery), while the remaining 26 were classified as optimal.

With regard to the average discrimination rate, the division of the subjects was quite balanced, with 13 subjects in the excellent category (Communication Skills, Legal Medicine, Emergencies, Palliatives, Oncology, Geriatrics, Plastic Surgery, Bioethics, Anesthesiology, Psychiatry, Rheumatology, Pediatrics, and Traumatology), nine subjects that could be considered good (Gynecology and Obstetrics, Neurology, Nephrology, Preventive, Ophthalmology, Clinical Management, Pneumology, Maxillofacial Surgery, and Hematology), and ten others that should be reviewed (Infectious Diseases, Otolaryngology, Endocrinology, Digestive, Immunology, Cardiology, Genetics, Physiology, Biochemistry, and Pharmacology). Finally, four subjects were classified as poorly discriminated (Dermatology, Vascular Surgery, Pathological Anatomy, and Anatomy).

As far as discrimination measured with the average of the point biserial correlation coefficient is concerned, only two subjects were considered excellent (Bioethics and Genetics), eleven showed good discrimination (Oncology, Ophthalmology, Communication Skills, Anesthesiology), Hematology, Endocrinology, Otolaryngology, Pneumology, Immunology, Palliative Care, and Cardiology), seven subjects were classified as poor (Dermatology, Emergencies, Plastic Surgery, Anatomy, Vascular Surgery, Geriatrics, and Pathological Anatomy) and the remaining 16 subjects were classified as regular.

On the other hand, for the discrimination index calculated according to the IRT, the average value of discrimination was 0.677, which falls into the regular category, with a standard deviation of 0.443.

For the difficulty index calculated according to the IRT, whose values are presented in the same table, there were no commonly accepted cut-off points like those of the previous coefficients that allowed us to classify the results obtained by subject. It should be noted that the mean value of the difficulty coefficient of all the examination questions was –0.389, with a standard deviation of 13.143.

### 3.3. Analysis of the Exam Questions Grouped by Logs or Blocks

The 36 subjects were grouped into three trunks or blocks, as shown in Table 2 and Table 3. Table 4 shows the values of the difficulty indices, difficulty with correction of the random effects, discrimination, and point biserial correlation index in each block of questions. For the three blocks of subjects considered, the average of the values of the difficulty index were quite similar with a maximum difference between groups of 0.102. In the case of the difficulty index with correction for random effects, the maximum difference was 0.127.

In the classification relative to the average of the discrimination index, there were differences in interest between the three blocks into which the questions were divided, with the most discriminatory block being that of Other specialties, followed by Systems and Basic science. On the other hand, the averages of the values of the point biserial correlation index for the three blocks were very similar.

### 3.4. Analysis by Question Type

Following the criteria used in previous publications [9,11,12], the questions were classified into four categories: clinical cases, clinical cases with image, negative questions, and test questions.

Table 5 shows the results of the indicators analyzed for each of the question types. In both the difficulty index and the difficulty index with correction of the random effects, the questions regarding clinical cases with an image were of greatest difficulty, followed by test questions, then negative questions, and finally, clinical cases without an image.

The discrimination index shows that the test questions were the most discriminative questions, followed by negative questions, clinical cases, and finally, clinical cases with an image. From the point of view of the point biserial correlation coefficient, the questions with the highest value in this index were the negative questions, followed by clinical cases, test cases, and finally, clinical cases with an image. For the discrimination coefficient calculated according to IRT, the resulting order of classification was the same.

The IRT difficulty index was also calculated for the examination questions, divided both by blocks and by question type. The values obtained for the different blocks were as follows: Systems −3.142 (12.560), Basic science 0.869 (5.801), and Other specialties 2.311 (14.590). For the questions divided by type, the order was as follows: clinical case with image −2.802 (17.640), clinical case −0.865 (4.801), test 0.156 (22.021), and negative 1.809 (13.890).

## 4. Discussion

With regard to the limitations of this study, it should be noted that unlike the publications produced by the Spanish Ministry of Health [10], which provide information on all those examined in each call, only 3868 people who took the MIR test were available for this study. Note that the most recent work on the psychometry of the MIR questions published by the Ministry dates back to 1993.

In addition, it should be borne in mind that the sample in this study was made up of people who entered their examination answers into the web application. This represented 27.31% of the total number of doctors who took the exam, but we are aware of the selection bias because self-selection was involved. Note that the median of the net points for the doctors in the sample was 119.67, while that of the overall population, according to the results of the Ministry’s lists of results, was 102.83. Finally, in our analysis, we carried out a quantitative study of the questions only, without going into how they were written [15]. A strength of this study is that we analyzed the test by combining CTT with IRT, and another strength is the size of the available sample.

The average value of the difficulty index of the 2018 test was 0.629, a very similar result to that of the average of the tests taken between 2009 and 2017 [12]. The average difficulty index with correction for the effects of chance had a value of 0.515, lower than the 0.5552 average of the tests from 2009 to 2017 [12]. In this case, the exam with the closest value was that of 2010 with a value of 0.5142 [12].

The average discrimination index of all test questions was 0.277, which was below the average from 2009 to 2017, which was 0.3203, although it was an improvement on that of the tests of 2016 (0.2552) and 2017 (0.2407).

Furthermore, the average of the point biserial correlation coefficient presented an average value of 0.275 in the 2018 test, which is lower than the average from 2009 to 2017 [12], although it is slightly better than the values of the 2016 and 2017 tests (0.2693 and 0.2556, respectively), which have the values closest to those of the 2018 examination.

The average value of the difficulty of the test calculated according to the two-parameter model of the IRT was greater than the average difficulty of the test from 2010 to 2017 [12]. Thus, in the case of the 2018 examination, the average difficulty value was –0.384, while the average difficulty values of the time series of tests analyzed was −0.7692 [12]. The 2018 MIR test, evaluated by this metric, is therefore the second most difficult in the 10-year time series. Please note that the values of the IRT parameters considered for this comparison were obtained for the test of each year using the available sample and without performing any kind of calibration, as there was neither a common sample nor common items in these years.

With regard to the average value of discrimination, which was also calculated following the model of two parameters of the IRT, the result obtained was 0.677, which is somewhat lower than the average of the tests taken between 2009 and 2017 (0.7617) [12] even though it is in fact, a result very close to that of the 2017 exam.

## 5. Conclusions

The results show a slight reduction in the values of discrimination in the last three calls. From our point of view, this fact may be related to the presence in these calls of a greater number of recent Spanish graduates, most of whom completed the test to gain access to specialized health training (MIR) in the same year as they completed their studies of Medicine. This subset may have very similar levels of knowledge to each other, which would make it difficult to discriminate between them through the test questions. This hypothesis could be confirmed or refuted if the analysis were carried out of all those taking the test segmented by year of graduation and nationality; however, this data is available only to the Spanish Ministry of Health.

### Limitations and Recommendations

One of the main limitations of this study is that we did not analyze the information of all the exam candidates, only a subset of them, which in the case of the 2018 exam represented 27.31% of the total. This database cannot be considered as a random sample of the total population as it has a bias. The bias is due to the higher probability that students with higher marks will voluntarily enter their answers/results into the web application. Although the methodology we employed was the same as that used in the all years under comparison, it did not allow us to obtain a complete picture of the MIR test because the profiles of exam candidates that took part in this study do not exactly match those of the overall population. However, despite these limitations, we consider that this research is of interest as the profiles of those candidates who entered their exam results into the website will remain stable and be available in the future, and also, as far as it is known by the authors, there are no other studies on MIR exams with either a bigger or an unbiased sample that have been performed since the one [16] that analyzed the results of exams in 2005 and 2006, and which was published in the Spanish language. Another possible limitation of this study is that we only performed a quantitative psychometric analysis, without taking into account any qualitative factors relating to the questions, for example, how they are written, what kind of words are employed, etc.

As we have indicated previously, it would be interesting to repeat this analysis using the answers of all examinees and not only those who entered their answers into the web application, thereby constituting a non-random sample. In order to make this possible, we encourage the ministries in charge of the test to make publicly available the anonymized information about the answers of all individuals to each question of the test, so that researchers can use them.

Finally, we believe that the use of this information by the members of the technical committee would also be of interest. This is because, in our opinion, analysis of the psychometric performance of the questions prior to the cancellation process by the technical committee, would improve such a process, as it would not only take into account the number of claims presented for each question or how the questions are written but would also consider the psychometric parameters of the questions.

## Figures and Tables

**Table 1:** Valuation in categories of difficulty indices, difficulty index with random effects correction, discrimination index, and point biserial correlation index.

Difficulty Index		Difficulty Index with Random Effects Correction		Discrimination Index		Correlation Index Point Biserial		Discrimination Index IRT	
Values	Categories	Values	Categories	Values	Categories	Values	Categories	Values	Categories
>0.8	Easy	>0.8	Very easy	>0.34	Excellent	>0.39	Excellent	>1	Excellent
>0.6 to 0.8	Acceptable	>0.66 to 0.80	Easy	>0.24 to 0.34	Good	>0.30 to 0.39	Good	>0.70 to 1	Good
>0.5 to 0.6	Excellent	>0.33 to 0.66	Excellent	>0.14 to 0.24	Revisable	>0.20 to 0.30	Regular	>0.40 to 0.70	Regular
>0.3 to 0.5	Acceptable	>0 to 0.33	Difficult	0 to 0.14	Bad	0–0.20	Poor	0–0.40	Poor
0–0.3	Difficult	–0.33 to 0	Very difficult	<0	Very bad	<0	Lousy	<0	Lousy

**Table 2:** Difficulty index, difficulty index with random effects correction, and discrimination index.

Block	Subject	Number of Questions	Difficulty Index	Difficulty Index with Random Effects Correction	Discrimination Index
**Systems**	Digestive	16	0.679 (0.276)	0.577 (0.366)	0.209 (0.098)
	Cardiology	15	0.732 (0.155)	0.648 (0.206)	0.170 (0.127)
	Infectious Diseases	14	0.514 (0.294)	0.360 (0.394)	0.238 (0.118)
	Nephrology	13	0.671 (0.226)	0.566 (0.298)	0.309 (0.008)
	Endocrinology	12	0.643 (0.253)	0.535 (0.326)	0.229 (0.107)
	Pneumology	11	0.706 (0.182)	0.614 (0.239)	0.289 (0.084)
	Neurology	10	0.722 (0.143)	0.641 (0.191)	0.320 (0.129)
	Haematology	10	0.652 (0.209)	0.545 (0.268)	0.255 (0.004)
	Rheumatology	6	0.656 (0.168)	0.545 (0.225)	0.398 (0.003)
**Basic**	Genetics	6	0.816 (0.072)	0.766 (0.088)	0.169 (0.016)
	Immunology	5	0.528 (0.177)	0.388 (0.225)	0.182 (0.001)
	Anatomy	5	0.482 (0.279)	0.319 (0.373)	0.021 (0.120)
	Pharmacology	5	0.529 (0.173)	0.380 (0.232)	0.157 (0.003)
	Pathological Anatomy	4	0.376 (0.204)	0.197 (0.238)	0.075 (0.086)
	Biochemistry	1	0.486	0.369	0.162
**Other**	Preventive	17	0.588 (0.235)	0.466 (0.311)	0.309 (0.079)
	Gynaecology and Obstetrics	12	0.613 (0.198)	0.492 (0.265)	0.328 (0.110)
	Paediatrics	10	0.581 (0.271)	0.452 (0.357)	0.378 (0.063)
	Psychiatry	7	0.725 (0.134)	0.642 (0.181)	0.409 (0.072)
	Traumatology	7	0.628 (0.144)	0.514 (0.192)	0.376 (0.102)
	Clinical Management	5	0.524 (0.321)	0.378 (0.422)	0.290 (0.136)
	Emergency	5	0.602 (0.222)	0.475 (0.295)	0.480 (0.032)
	Ophthalmology	4	0.751 (0.099)	0.673 (0.130)	0.296 (0.108)
	Dermatology	4	0.407 (0.034)	0.221 (0.046)	0.130 (0.087)
	Physiology	3	0.682 (0.067)	0.597 (0.082)	0.169 (0.001)
	Otorhinolaryngology	3	0.709 (0.275)	0.621 (0.358)	0.229 (0.080)
	Oncology	3	0.568 (0.134)	0.433 (0.177)	0.456 (0.003)
	Palliatives	3	0.593 (0.318)	0.461 (0.423)	0.466 (0.006)
	Geriatrics	3	0.348 (0.245)	0.140 (0.333)	0.442 (0.005)
	Maxilo-Facial Surgery	2	0.658 (0.067)	0.551 (0.092)	0.269 (0.002)
	Plastic Surgery	2	0.658 (0.084)	0.573 (0.079)	0.427 (0.005)
	Anaesthesiology	2	0.844 (0.073)	0.798 (0.095)	0.420 (0.001)
	Vascular Surgery	2	0.297 (0.231)	0.075 (0.317)	0.090 (0.066)
	Bioethics	1	0.941	0.922	0.421
	Communication Skills	1	0.971	0.964	0.482
	Legal Medicine	1	0.667	0.568	0.481
	**Total**	**230**	**0.629 (0.226)**	**0.515 (0.297)**	**0.277 (0.130)**

**Table 3:** Point biserial correlation and difficulty and discrimination indexes according to IRT.

Block	Subject	Number of Questions	Point Biserial Correlation	Difficulty IRT	IRT Discrimination
**Systems**	Digestive	16	0.246 (0.160)	−8.065 (23.309)	0.629 (0.508)
	Cardiology	15	0.305 (0.130)	−1.860 (1.147)	0.787 (0.482)
	Infectious Diseases	14	0.249 (0.213)	−6.094 (23.097)	0.642 (0.763)
	Nephrology	13	0.267 (0.096)	−0.685 (3.268)	0.655 (0.348)
	Endocrinology	12	0.314 (0.121)	−0.959 (2.493)	0.794 (0.357)
	Pneumology	11	0.283 (0.132)	−3.974 (7.777)	0.704 (0.416)
	Neurology	10	0.311 (0.082)	−1.634 (1.233)	0.758 (0.294)
	Haematology	10	0.339 (0.121)	−0.739 (1.657)	0.856 (0.459)
	Rheumatology	6	0.281 (0.121)	−1.016 (0.945)	0.686 (0.438)
**Basic**	Genetics	6	0.395 (0.080)	−1.892 (0.334)	1.067 (0.374)
	Immunology	5	0.311 (0.117)	0.194 (1.582)	0.679 (0.321)
	Anatomy	5	0.176 (0.078)	1.291 (4.446)	0.368 (0.125)
	Pharmacology	5	0.257 (0.067)	−0.188 (1.719)	0.539 (0.143)
	Pathological Anatomy	4	0.145 (0.156)	6.849 (13.448)	0.315 (0.337)
	Biochemistry	1	0.257	0.066	0.536
**Other**	Preventive	17	0.271 (0.099)	−0.329 (2.449)	0.635 (0.259)
	Gynaecology and Obstetrics	12	0.236 (0.106)	0.83 (6.826)	0.515 (0.273)
	Paediatrics	10	0.239 (0.139)	6.311 (20.638)	0.604 (0.447)
	Psychiatry	7	0.279 (0.119)	−1.780 (0.97)	0.661 (0.515)
	Traumatology	7	0.294 (0.082)	−0.930 (1.043)	0.617 (0.221)
	Clinical Management	5	0.258 (0.134)	0.726 (3.661)	0.685 (0.407)
	Emergency	5	0.197 (0.116)	1.264 (7.176)	0.391 (0.257)
	Ophthalmology	4	0.375 (0.158)	−1.677 (0.372)	0.991 (0.599)
	Dermatology	4	0.198 (0.073)	1.863 (2.38)	0.374 (0.187)
	Physiology	3	0.255 (0.034)	−1.755 (0.853)	0.503 (0.061)
	Otorhinolaryngology	3	0.313 (0.123)	−1.656 (1.883)	1.107 (0.978)
	Oncology	3	0.385 (0.099)	−0.320 (0.696)	0.959 (0.386)
	Palliatives	3	0.307 (0.096)	−0.453 (2.396)	0.733 (0.227)
	Geriatrics	3	0.152 (0.203)	57.036 (51.707)	0.304 (0.505)
	Maxilo-Facial Surgery	2	0.289 (0.051)	−1.260 (0.281)	0.5814 (0.157)
	Plastic Surgery	2	0.193 (0.005)	−1.936 (1.202)	0.376 (0.035)
	Anaesthesiology	2	0.369 (0.067)	−2.178 (0.143)	0.997 (0.358)
	Vascular Surgery	2	0.164 (0.162)	11.891 (16.472)	0.338 (0.366)
	Bioethics	1	0.481	−2.270	2.006
	Communication Skills	1	0.372	−2.908	1.887
	Legal Medicine	1	0.270	−1.482	0.518
	**Total**	**230**	**0.275 (0.129)**	**−0.389 (13.143)**	**0.677 (0.443)**

**Table 4:** Difficulty index, difficulty index with random effects correction, discrimination index, biserial correlation index, and discrimination index according to the IRT of the questions of the MIR exam of the year 2018 for each block of questions. Average values, standard deviations, and classification into categories are shown.

		**Difficulty Index (Categories)**					
	**Difficulty Index**	**Easy**	**Acceptable**	**Optimal**	**Difficult**	**Total**	
**Basic**	0.561 (0.227)	3	16	4	3	26	
**Systems**	0.663 (0.226)	37	49	9	12	107	
**Other**	0.609 (0.221)	19	57	9	12	97	
**Total**	0.629 (0.226)	59	122	22	27	230	
		**Difficulty index with correction of random effects (categories)**					
	**Difficulty index with random effects correction**	**Very easy**	**Easy**	**Optimal**	**Difficult**	**Very difficult**	**Total**
**Basic**	0.431 (0.295)	3	4	11	6	2	26
**Systems**	0.558 (0.299)	25	24	36	13	9	107
**Other**	0.489 (0.292)	14	21	33	21	8	97
**Total**	0.515 (0.297)	42	49	80	40	19	230
		**Discrimination index (categories)**					
	**Discrimination Index**	**Excellent**	**Good**	**Revisable**	**Bad**	**Very bad**	**Total**
**Basic**	0.126 (0.085)	0	0	20	2	4	26
**Systems**	0.256 (0.109)	11	49	31	16	0	107
**Other**	0.342 (0.118)	60	22	6	9	0	97
**Total**	0.277 (0.129)	71	71	57	27	4	230
		**Point biserial correlation index (categories)**					
	**Point biserial correlation index**	**Excellent**	**Good**	**Regular**	**Poor**	**Too bad**	**Total**
**Basic**	0.267 (0.129)	5	5	7	9	0	26
**Systems**	0.286 (0.139)	23	23	37	22	2	107
**Other**	0.267 (0.117)	11	28	29	29	0	97
**Total**	0.275 (0.128)	39	56	73	60	2	230
		**Discrimination index IRT (categories)**					
	**Discrimination Index IRT**	**Excellent**	**Good**	**Regular**	**Poor**	**Too bad**	**Total**
**Basic**	0.620 (0.377)	27	18	42	15	5	107
**Systems**	0.719 (0.471)	4	6	7	9	0	26
**Other**	0.646 (0.427)	11	25	35	26	0	97
**Total**	0.677 (0.443)	42	49	84	50	5	230

**Table 5:** Index of difficulty, index of difficulty with random effects correction, index of discrimination, index of point biserial correlation, and index of discrimination according to the IRT of the questions of the year 2018 exam by type of question. Average values, standard deviations, and classification into categories are shown.

		**Difficulty index (categories)**					
	**Difficulty index**	**Easy**	**Acceptable**	**Optimal**	**Difficult**	**Total**	
**Clinical Cases**	0.662 (0.216)	32	60	10	9	111	
**Clinical cases with image**	0.561 (0.244)	7	15	5	5	32	
**Negative**	0.645 (0.224)	15	26	3	6	50	
**Test**	0.568 (0.221)	5	21	4	7	37	
**Total**	0.629 (0.226)	59	122	22	27	230	
		**Difficulty index with random effects correction (categories)**					
	**Difficulty index with random effects correction**	**Very easy**	**Easy**	**Optimal**	**Difficult**	**Very difficult**	**Total**
**Clinical Cases**	0.557 (0.286)	26	25	37	16	7	111
**Clinical cases with image**	0.426 (0.323)	4	5	11	8	4	32
**Negative**	0.535 (0.294)	10	12	15	10	3	50
**Test**	0.437 (0.293)	2	7	17	6	5	37
**Total**	0.515 (0.297)	42	49	80	40	19	230
		**Discrimination index (categories)**					
	**Discrimination Index**	**Excellent**	**Good**	**Revisable**	**Bad**	**Very bad**	**Total**
**Clinical Cases**	0.278 (0.127)	34	34	30	10	3	111
**Clinical cases with image**	0.225 (0.117)	6	9	8	9	0	32
**Negative**	0.289 (0.112)	18	17	12	3	0	50
**Test**	0.302 (0.159)	13	11	7	5	1	37
**Total**	0.277 (0.129)	71	71	57	27	4	230
		Point biserial correlation index (categories)					
	**Point biserial correlation index**	**Excellent**	**Good**	**Regular**	**Poor**	**Too bad**	**Total**
**Clinical Cases**	0.291 (0.131)	24	33	28	24	2	111
**Clinical cases with image**	0.188 (0.087)	1	1	13	17	0	32
**Negative**	0.305 (0.126)	14	8	18	10	0	50
**Test**	0.263 (0.122)	7	7	14	9	0	37
**Total**	0.275 (0.128)	46	49	73	60	2	230
		**Discrimination index IRT (categories)**					
	**Discrimination Index IRT**	**Excellent**	**Good**	**Regular**	**Poor**	**Too bad**	**Total**
**Clinical Cases**	0.729 (0.471)	23	32	31	22	3	111
**Clinical cases with image**	0.416 (0.243)	1	1	15	14	1	32
**Negative**	0.783 (0.483)	14	7	22	7	0	50
**Test**	0.599 (0.335)	4	9	16	7	1	37
**Total**	0.677 (0.443)	42	49	84	50	5	230
